# Diabetic Retinopathy in Primary Healthcare in a Brazilian Municipality: A Cross‐Sectional Study Using Teleophthalmology

**DOI:** 10.1002/hcs2.70076

**Published:** 2026-05-11

**Authors:** Lorrana Luysse dos Anjos Assis, Fernando Korn Malerbi, Rosiane Chiaroti, Lívia Maria Ferrante Vizzotto Consoli, Vanessa Patrícia Pereira Motozo, Francisco Barbosa‐Junior, Rinaldo Eduardo Machado de Oliveira

**Affiliations:** ^1^ Ribeirão Preto Medical School University of São Paulo Ribeirão Preto São Paulo Brazil; ^2^ Department of Ophthalmology and Visual Sciences São Paulo Brazil; ^3^ Faculty of Health Sciences and Technologies University of Brasília Brasília Brazil

**Keywords:** diabetes complications, diabetes mellitus, digital health, health surveys, ophthalmology, public health, telemedicine

## Abstract

**Background:**

Diabetic retinopathy is a complication of diabetes and the leading cause of irreversible blindness in people of working age. This study aims to estimate the prevalence of diabetic retinopathy among individuals taking insulin who attend the primary healthcare in the municipality of Jardinópolis, state of São Paulo, Brazil.

**Methods:**

This is a cross‐sectional study with a sample of 152 participants, in which data collection occurred in March 2023 by means of interviews, laboratory exams and fundoscopy with a portable retinal camera mounted onto a device attached to a smartphone. The captured images were automatically analysed by means of deep learning algorithms for grading retinal alterations, as well as diabetic retinopathy.

**Results:**

The prevalence of diabetic retinopathy was estimated to be 28.3% (95% CI 21.1–35.4), with most of the cases corresponding to mild non‐proliferative diabetic retinopathy (65.1%). No association was found between diabetic retinopathy and the variables investigated. Concordance test between self‐reported and diagnosed diabetic retinopathy showed a Kappa coefficient of 0.30 (95% CI, 0.13–0.47), in which 16.5% of the participants reported having no diabetic retinopathy despite being diagnosed as having it. However, 9.9% of the participants self‐reporting having diabetic retinopathy were not diagnosed with it.

**Conclusions:**

The prevalence of diabetic retinopathy is within the national range. The lack of knowledge on the diagnosis of diabetic retinopathy and aspects related to such a complication of diabetes was found to be worrying. Therefore, one highlights the importance of primary healthcare programmes in promoting integral care to people with diabetes, including ocular health.

## Graphical abstract

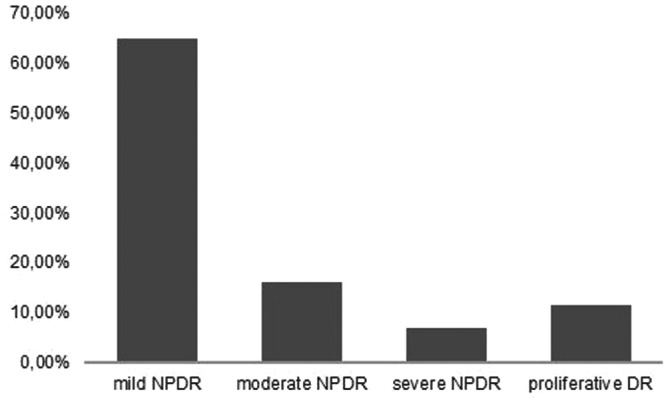

**Abbreviations**
CI: confidence interval
DR: diabetic retinopathy
FHU: family health unit
NPDR: non‐proliferative diabetic retinopathy
PDR: proliferative diabetic retinopathy
PHC: primary healthcare
PR: prevalence ratio
SD: standard deviation
SP: São Paulo
T1D: type 1 diabetes
T2D: type 2 diabetes

## 1 Background

Diabetic retinopathy (DR) is a microvascular complication in the retina of people with diabetes which, if not treated, can lead to definitive blindness [1]. A Brazilian study estimated a prevalence of DR at 35.7% among individuals with type 1 diabetes (T1D) [2], whereas another reported 37.3% among individuals with type 2 diabetes (T2D) [3]. A systematic review with meta‐analysis, in turn, estimated a prevalence of DR at 36.28% among people with diabetes, being more common in those suffering longer with the disease and living in the southern region of Brazil [4]. From this scenario, one can recognise that DR is a major public health problem deserving to be highlighted in the healthcare portfolio of services for people with diabetes.

In Brazil, primary healthcare (PHC) is the entry door for patients with non‐communicable chronic conditions, including diabetes, in which inter‐sectorial actions are proposed [5]. A study using data from a programme for improvement of access and quality in primary healthcare, conducted in the years of 2012, 2014 and 2017, showed that there was an improvement in the indicators related to care, structure and laboratory tests, except cases of diabetic foot. In addition, negative results were observed in the Northern Brazil regarding other regions. Among the gaps in the comprehensiveness of care for people with diabetes in the public healthcare system, there is yet no programme aimed at screening for DR in many municipalities [6].

It is known that vision loss may not be a usual event in the initial stages of DR. Therefore, ophthalmologic screening for early detection and immediate treatment is essential to prevent impairment as well as to reduce health costs [7]. Full ophthalmologic exam is the classical screening method, which is performed by means of colour retinography or retinal mapping [1]. However, the equipment used is often scarce in the public healthcare services, meaning that teleophthalmology based on fundoscopy for diagnosis of DR has been appropriate and cost‐effective [8]. A comparative prospective study performed in two reference health centres in the state of Minas Gerais, Brazil, showed evidence of cost reduction of US$ 28.76 per patient, with a calculated balance point of 12 exams/month or 1344 exams/year. It was also observed that teleophthalmology allowed the restrained demand for fundoscopy to be solved by means of early diagnosis and referral to other services when necessary [9].

DR represents a strong indicator of unfavourable clinical outcome, in addition to the financial impact. The screening model for DR using portable device and teleophthalmology can potentially increase the coverage in the PHC, thus optimising the care for diabetic patients and reducing the overall burden of an irreversible visual damage [10, 11]. Furthermore, studies have shown that DR may be associated with inadequate glycaemic control, as well as long‐standing diabetes, especially among people using insulin [1, 2]. It is primordial to carry out studies capable of presenting the panorama of DR in the PHC of small‐sized municipalities, which are the great majority in Brazil, to subside understanding, reflection and production of evidence for elaboration and implementation of viable programmes for prevention, diagnosis and treatment of DR. In this sense, the objective of the present study is to estimate the prevalence of DR among individuals taking insulin who attended the PHC of the municipality of Jardinópolis, São Paulo (SP), Brazil.

## 2 Methods

This is a cross‐sectional study performed in the municipality of Jardinópolis in the state of SP, Brazil. In 2022, the population was estimated in 45,282 people, with a development index of 0.735, classified as high based on longevity, education, and income data [12]. The present study integrates the programme called DIABETES Jardinópolis, which is a survey on the health of people with diabetes taking insulin who attend the local PHC, and whose data were collected in March of 2023.

In the sample size calculation, the reference population was of 834 people with diabetes on insulin for a prevalence of 13.37% of DR in South America [13], tolerable absolute error of 5% and 95% confidence interval (CI), yielding 152 participants. Male and female individuals were eligible for study, all aged above 18 years old, diagnosed with diabetes and enroled in the public healthcare services of the municipality. Individuals discontinuing the use of insulin for medical advice 7 days prior to the study, pregnant women, bedridden and/or domiciled people, individuals with acute infectious process and those with history of surgery or hospitalisation up to 3 months prior to the study were excluded. The participation of the individuals in this study was based on probabilistic sampling. A list of eligible study participants was compiled, and then a random drawing was performed. If the selected individual declined to participate, a new random drawing was performed. This continued until the calculated sample size was reached.

Data were obtained by means of interview, blood and urine exams, and ophthalmologic exam for DR. The instrument for data collection was a structured form divided into five blocks: (1) socio‐demographic and economic variables; (2) care of health; (3) access to and use of healthcare services; (4) use of medications for diabetes and adherence to insulin therapy; and (5) exam results (e.g., glycated haemoglobin, microalbuminuria and DR). One can emphasise that in the block on care of health the question was “Do you have or had DR caused by diabetes”.

Initially, in the ophthalmologic exam for DR, 5 mg/mL of proxymetacaine eye drops were dripped on each eye, followed by a drop of tropicamide 1% mydriatic eyes drops. Then, a portable retinal camera (Eyer, Phelcom Technologies, São Carlos, SP, Brazil) were used by a physician or nurse trained for the study. The captured images were automatically analysed by deep learning algorithms retinal alteration score and DR alteration score, consisting of convolutional neural networks trained on EyePACS data sets and fine‐tuned using data sets of portable device fundus images. Previous study has shown that the sensitivity for detection of DR was 90.48% (95% CI: 85.99–94.46), with specificity of 90.65% (95% CI: 84.54–94.93) [14]. The protocol for image acquisition was the following: two images of the fundus, one centred on the macula, one centred on the optic nerve and an additional image of the anterior segment. Next, the algorithm generated a heat map evidencing suspect changes in the retina with a colour scale ranging from blue to red, which corresponded to the variation of low and high importance, respectively [3]. All images were then revised by a previously trained ophthalmologist, who also issued a medical report.

The criteria of classification used were the following: absence of DR, mild non‐proliferative DR (NPDR), moderate NPDR, severe NPDR, very severe NPDR and proliferative DR (PDR), as established by the Brazilian Society of Diabetes [1]. In the case of absence of DR, the participant was instructed to undergo a fundus examination every year. Mild and moderate cases of non‐proliferative DR were referred to the municipal outpatient ophthalmology service for follow‐up. The cases of severe non‐proliferative DR, proliferative DR and DR with macular oedema were referred with high priority to the healthcare service for treatment with a retinal specialist.

The independent variables analysed were the following: sex, age (the absolute frequency was obtained and categorised as: below 45; 45–59; 60–74 and 75 or above), colour/race (White and Non‐White), education level (0; 1–9 years; 10–12 years; and 13 or more years of study), social‐economic group (A/B, C and D/E) [15], good or very health self‐perception (yes/no), abusive consumption of alcohol measured by AUDIT‐C (yes/no) [16], smoking habit (yes/no), type of diabetes (1; 2; don't know), duration of diabetes (the absolute frequency was obtained and categorised as “1 to 10 years”; “11 to 20 years”; “above 20 years”; “don't know”), self‐reported diagnosis of systemic arterial hypertension (yes/no; don't know), renal disease (yes/no; don't know), overweight/obesity (yes/no; don't know), dyslipidaemia (yes/no; don't know), ulcer/wound (yes/no; don't know), amputation (yes/no; don't know), access to private health insurance (yes/no), time elapsed since the last medical visit for treatment of diabetes (less than 6 months; 6 months or more), follow‐up of diabetes at the family health unit (FHU) (yes/no), adherence to insulin therapy (yes/no) [17], glycaemic control with glycated haemoglobin below 7% (53 mmol/mol) (yes/no) [18] and albuminuria (normal [< 30 mg/g]); microalbuminuria (30–299 mg/g) and macroalbuminuria (> 300 mg/g) [19]. It should be noted that venous blood and urine were collected in this study.

A pilot test was conducted with 10% of the calculated sample (*n* = 15) to analyse the research instrument and identify operational problems and difficulties. Participants from this stage were not included in the final sample.

Statistical analysis was performed by using software SAS 9.4. Initially, data were described by means of absolute and percentage frequencies (qualitative variables) and by using centralisation measures and dispersion such as mean, standard deviation (SD), minimum, median and maximum (quantitative variables). The Poisson regression model with robust variance was used to estimate the prevalence ratio (PR) and its prospective 95%CI of DR among the variables of interest, crudely and adjusted for sex, age group and duration of diabetes. Multiple comparisons between categories of independent variables with more than two levels were performed using contrasts of estimated marginal means (LS‐means) with Bonferroni adjustment to control type I error, as recommended for generalised models with binary outcomes. The significance level of *p* < 0.05 was used. For analysis of concordance between self‐reported DR and diagnosis of DR, Kappa coefficient test was used because it measures the degree of concordance between tests when variables are categorical. If the Kappa coefficient has a maximum value of 1, then it corresponds to a perfect concordance, whereas a value of 0 indicates that the concordance is equal to that expected by chance.

The present study was approved by the Research Ethics Committee of the Dr. Joel Domingos School Health Centre of the Ribeirão Preto Medical School of the University of São Paulo following the recommendations of the National Research Ethics Commission and according to protocol number 466/2012. The Certificate of Presentation for Ethical Review was obtained with the number 64301322.0.0000.5414. All the participants of the study freely signed an informed consent form.

## 3 Results

A total of 152 people with diabetes taking insulin participated in this study according to the calculated sample. It was noted that 12% of eligible people did not want to participate, so new draws were carried out to obtain the sample. Most of this sample was female (63.2%), with median age of 60 years old (minimum of 28 years and maximum of 86 years), self‐reporting to be Non‐White (61.2%), having studied for 1–9 years (63.2%) and belonging to social‐economic class C (67.8%). Among the participants who knew the type of diabetes they had (*n* = 111), 91% reported it to be type 2. The mean duration of diabetes was 17.1 years (SD = 10.1). The majority had no private health insurance (84.2%), thus having their diabetes followed up at FHU (82.9%) and whose last medical visit had been within the last 6 months (86.2%). It was found that the majority did not adhere to the insulin therapy (52.7%), nor were under glycaemic control (77%), with most having normal albuminuria (56.6%) (Table 1). The median value of glycated haemoglobin was 8.7% (72 mmol/mol), ranging from 5.2% to 17.6%, whereas the median value of albuminuria was 21.8 mg/g, ranging from 2.3 to 7500 mg/g.

**Table 1:** Characteristics of the sample and prevalence of DR according to socio‐demographic, economic, clinic variables and use of health services. Diabetes Jardinópolis survey, SP, Brazil, 2023 (*n* = 152).

Variables	Sample *n* (%)	Prevalence of DR (95% CI)
Sex		
Female	96 (63.2)	25.0 (16.3–33.7)
Male	56 (36.8)	34.0 (21.5–46.3)
Age (years)		
< 45	18 (11.8)	11.1 (0–25.3)
45–59	57 (37.5)	31,6 (19.5–43.6)
60–74	60 (39.5)	25.0 (14.1–35.9)
≥ 75	17 (11.2)	47.1 (23.3–70.8)
Colour/race (self‐reported)		
Non‐White	93 (61.2)	31.2 (21.8–40.6)
White	59 (38.8)	23.7 (12.9–34.6)
Education level (school years)		
0	21 (13.8)	38.1 (17.3–58.9)
1–9	96 (63.2)	29.2 (20.1–38.2)
10–12	28 (18.4)	21.4 (6.2–36.6)
≥ 13	7 (4.6)	14.3 (0–40.2)
Socio‐economic group [15]		
A/B	15 (9.9)	20.0 (0–40.2)
C	103 (67.8)	27.2 (18.6–35.8)
D/E	34 (22.3)	35.3 (19.2–51.4)
Self‐reported health		
Negative	77 (50.7)	27.3 (17.3–37.2)
Positive	75 (49.3)	29.3 (19.0–39.6)
Abusive alcohol consumption [16]		
No	119 (78.3)	26.0 (18.2–33.9)
Yes	33 (21.7)	36.4 (19.9–52.8)
Smoking habit		
No	133 (87.5)	27.8 (20.2–35.4)
Yes	19 (12.5)	31.6 (10.7–52.5)
Self‐reported type of diabetes (*n* = 111)		
1	10 (9.0)	30.0 (1.6–58.4)
2	101 (91.0)	29.7 (20.8–38.6)
Duration of diabetes (years) (*n* = 148)		
1–10	47 (31.8)	19.1 (7.9–30.4)
11–20	57 (38.5)	33.3 (21.1–45.6)
> 20	44 (29.7)	34.1 (20.1–48.1)
Self‐report health condition		
Systemic arterial hypertension	108 (71.0)	28.7 (20.2–37.2)
Dyslipidaemia	88 (57.9)	26.1 (16.9–35.3)
Overweight/obesity	66 (43.4)	25.8 (15.2–36.3)
Renal disease	17 (11.2)	29.4 (7.7–51.1)
Ulcers/wound	18 (11.8)	33.3 (11.6–55.1)
Amputation	7 (4.6)	42.9 (6.2–79.5)
Private health insurance		
No	128 (84.2)	27.3 (19.9–35.1)
Yes	24 (15.8)	33.3 (14.5–52.2)
Medical visit within the last 6 months for treatment of diabetes		
Yes	131 (86.2)	29.0 (21.2–36.8)
No	21 (13.8)	23.8 (5.6–42.0)
Treatment of diabetes at a family health unit		
Yes	126 (82.9)	27.8 (19.9–35.6)
No	26 (17.1)	30.8 (13.0–48.5)
Adherence to insulin therapy [17]		
No	80 (52.7)	32.5 (22.2–42.8)
Yes	72 (47.3)	23.6 (13.8–33.4)
Glycaemic control [18]		
No	117 (77.0)	30.8 (22.4–39.1)
Yes	35 (23.0)	20.0 (6.7–33.2)
Albuminuria [19]		
Normal	86 (56.6)	22.1 (13.3–30.9)
Microalbuminuria	46 (30.3)	34.8 (21.0–48.5)
Macroalbuminuria	20 (13.1)	40.0 (18.5–61.5)

Abbreviations: CI, confidence interval; DR, diabetic retinopathy; SP, São Paulo.

**Figure 1:**
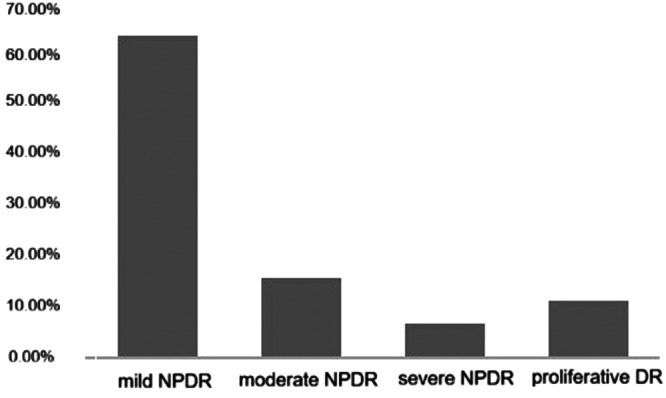
Classification of DR in the study sample. Diabetes Jardinópolis survey, SP, Brazil, 2023 (*n* = 43). DR, diabetic retinopathy; NPDR, non‐proliferative diabetic retinopathy; SP, São Paulo.

**Figure 2:**
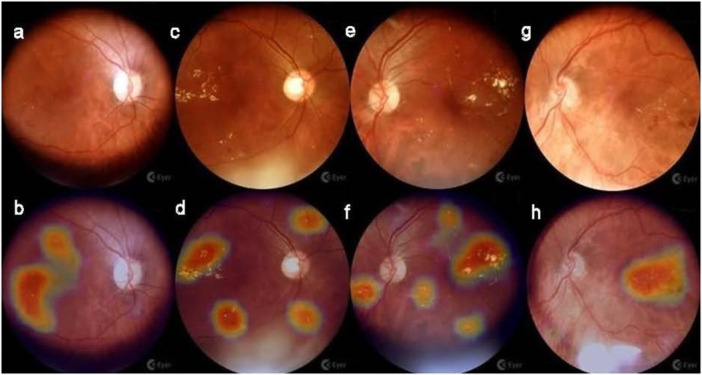
Types of DR identified in the participants and respective heat maps. (a) right eye with mild NPDR; (b) heat map of right eye with mild NPDR; (c) right eye with moderate NPDR; (d) heat map of right eye with moderate NPDR; (e) left eye with severe NPDR; (f) heat map of left eye with severe NPDR; (g) left eye with severe PDR; (h) heat map of left eye with severe PDR (Eyer, Phelcom Technologies, São Carlos, Brazil) [14]. DR, diabetic retinopathy; NPDR, non‐proliferative diabetic retinopathy; PDR, proliferative diabetic retinopathy.

The prevalence of DR was estimated in 28.3% (95%CI: 21.1–35.4), in which 65.1% of the cases corresponded to mild NPDR (Figure 1). Some images can be seen in Figure 2. No association between DR and the variables investigated was found (Table 2). However, among the individuals with DR, it was observed that the frequency of DR in those aged 75 year or older was of 47.1%. On the other hand, such a frequency was of 30.8% in those participants not under glycaemic control, as well as in those with macroalbuminuria (40%) and who were amputated due to diabetic complications (42.9%).

**Table 2:** Association of DR with socio‐demographic, economic, clinic variables and use of health services. Diabetes Jardinópolis survey, SP, Brazil, 2023 (*n* = 152).

Variables	Gross PR (95% CI)	*p*‐value	Adjusted PR^a^ (95% CI)	*p*‐value
Sex				
Female	0.74 (0.45–1.22)	0.23	0.70 (0.43–1.14)	0.15
Male	1.00		1.00	
Age (years)				
< 45	0.24 (0.04–1.56)	0.26	0.25 (0.04–1.67)	0.33
45–59	0.67 (0.29–1.57)	0.99	0.77 (0.31–1.90)	0.99
60–74	0.53 (0.22–1.31)	0.38	0.54 (0.21–1.39)	0.51
≥ 75	1.00		1.00	
Colour/race (self‐reported)				
Non‐White	1.00		1.00	
White	0.76 (0.44–1.32)	0.33	0.81 (0.48–1.37)	0.44
Education level (school years)				
0	2.67 (0.21–34.17)	0.99	3.09 (0.27–35.93)	0.99
1–9	2.04 (0.17–24.34)	0.99	1.96 (0.18–21.35)	0.99
10–12	1.50 (0.11–20.66)	0.99	1.67 (0.13–20.67)	0.99
≥ 13	1.00		1.00	
Socio‐economic class [15]				
A/B	0.57 (0.15–2.20)	0.57	0.52 (0.14–1.97)	0.72
C	0.77 (0.39–1.52)	0.63	0.78 (0.41–1.47)	0.99
D/E	1.00		1.00	
Self‐reported health				
Negative	0.93 (0.56–1.54)	0.78	1.00 (0.60–1.67)	0.99
Positive	1.00		1.00	
Abusive alcohol consumption [16]				
No	0.72 (0.42–1.23)	0.23	0.70 (0.40–1.23)	0.22
Yes	1.00		1.00	
Smoking habit				
No	0.88 (0.43–1.80)	0.73	1.01 (0.53–1.92)	0.99
Yes	1.00		1.00	
Self‐reported type of diabetes (*n* = 111)				
1	1.01 (0.37–2.73)	0.98	1.48 (0.62–3.52)	0.38
2	1.00		1.00	
Duration of diabetes (years) (*n* = 148)				
1–10	0.56 (0.23–1.35)	0.34	0.56 (0.23–1.39)	0.39
11–20	0.98 (0.51–1.92)	0.99	1.05 (0.53–2.05)	0.99
> 20	1.00		1.00	
Systemic arterial hypertension				
No	0.89 (0.49–1.61)	0.70	0.95 (0.54–1.68)	0.86
Yes	1.00		1.00	
Dyslipidaemia				
No	1.20 (0.72–1.98)	0.49	1.26 (0.78–2.04)	0.35
Yes	1.00		1.00	
Overweight/obesity				
No	1.17 (0.70–1.98)	0.55	1.19 (0.71–1.98)	0.51
Yes	1.00		1.00	
Renal disease				
No	0.96 (0.44–2.10)	0.91	1.10 (0.47–2.55)	0.82
Yes	1.00		1.00	
Ulcers/wound				
No	0.83 (0.41–1.68)	0.60	0.92 (0.43–2.00)	0.84
Yes	1.00		1.00	
Amputation				
No	0.64 (0.26–1.58)	0.33	0.71 (0.29–1.78)	0.47
Yes	1.00		1.00	
Private health insurance				
No	0.82 (0.44–1.54)	0.54	0.72 (0.39–1.34)	0.30
Yes	1.00		1.00	
Medical visit within the last 6 months for treatment of diabetes				
No	1.22 (0.54–2.74)	0.63	1.22 (0.57–2.62)	0.62
Yes	1.00		1.00	
Treatment of diabetes at a family health unit				
No	1.11 (0.58–2.10)	0.75	1.17 (0.64–2.17)	0.61
Yes	1.00		1.00	
Adherence to insulin therapy [17]				
No	1.38 (0.82–2.32)	0.23	1.36 (0.82–2.26)	0.23
Yes	1.00		1.00	
Glycaemic control [18]				
No	1.54 (0.75–3.15)	0.24	1.42 (0.72–2.81)	0.31
Yes	1.00		1.00	
Albuminuria [19]				
Normal	0.55 (0.24–1.25)	0.24	0.54 (0.25–1.17)	0.17
Microalbuminuria	0.87 (0.39–1.96)	0.99	0.77 (0.37–1.58)	0.99
Macroalbuminuria	1.00		1.00	

Abbreviations: CI, confidence interval; DR, diabetic retinopathy; PR, prevalence ratio; SP, São Paulo.

^a^ Adjusted for sex, age group and duration of diabetes.

Kappa concordance test between self‐reported and diagnosed DR had a coefficient of 0.30 (95%CI: 0.13–0.47), in which 16.5% of the participants reported having no DR despite being diagnosed as having it. However, 9.9% of the participants self‐reported DR and were not diagnosed with the disease (Table 3).

**Table 3:** Kappa coefficient between self‐reported and diagnosed DR. Diabetes Jardinópolis survey, SP, Brazil, 2023 (*n* = 152).

Self‐reported DR	Diagnosed DR(%)		
	No	Yes	Total
No	94 (61.8)	25 (16.5)	119 (78.3)
Yes	15 (9.9)	18 (11.8)	33 (21.7)
Total	109 (71.7)	43 (28.3)	152 (100.0)

*Note:* Kappa coefficient = 0.30 (95%CI: 0.13–0.47). Accuracy = 73.68% (95%CI: 66.68–80.68).

Abbreviations: CI, confidence interval; DR, diabetic retinopathy; SP, São Paulo.

In addition, by means of retinography, it was possible to identify alterations in the optic nerve in five participants, possibly due to glaucoma, drusen of the posterior pole (age‐related macular degeneration) in seven participants, cataract in two participants, posterior capsular opacification after cataract surgery in one participant, moderate hypertensive retinopathy in one participant, and signs of atherosclerosis in two participants. After these diagnoses, all the participants were instructed and referred to the municipal healthcare unit for ophthalmologic consultation.

## 4 Discussion

The estimated prevalence of DR in the PHC of a Brazilian small‐sized municipality was found to be higher than the global prevalence [13]. However, this figure is similar across the country [4]. The frequency of DR was higher among individuals not under glycaemic control, who did not adhere to insulin therapy, had macroalbuminuria and suffered amputation. In addition, it was noted that the diabetic participants who were not followed up at FHU also presented higher frequency of DR. One should point to the low concordance between self‐reported and diagnosed DR, which was verified with the use of a portable retinal camera, smartphone and AI algorithm.

A systematic review with metanalysis estimated that the global prevalence of DR was of 22.27% (95%CI: 19.73–25.03), being higher in Africa (35.90%), North America and Caribbean countries (33.30%), On the other hand, lower frequencies were observed in South and Central Americas (13.37%) [13]. Another systematic review with metanalysis found a similarity with this study in Brazil, whose prevalence varied from 7.6% to 44.4%. Such a wide interval was attributed to regional variations across the country and methods used in the studies included [4].

About the abusive alcohol consumption, different studies presented controversial results. There were studies reporting no evidence of association between abusive alcohol consumption and DR, whereas others showed possible relations. Due to the potential methodological limitations of the observational studies, the causal association between these variables and DR may be confounded with reverse causality regarding colour/race, sex and age. About smoking habits, other studies found an association between daily smoking and PDR [20].

This research did not observe an association between inadequate glycaemic control and DR, different from other studies. A limitation could have been the sample size. Two clinical randomised trials, namely, Diabetes Control and Complication Trial, which involved people with T1D, and the UK prospective diabetes study involving people with T2D, showed that a rigorous control of the glycaeted haemoglobin levels reduces the risk of development of DR and its progression. A systematic review with metanalysis and sequential analysis of clinical trials also suggested that intensive glycaemic control leads to a 20% reduction in the risk of DR [21].

It is known that kidney and eye, despite their functional differences, share similarities in the development paths and molecular structure, meaning that a severe renal compromise secondary to diabetic nephropathy is correlated to severe ocular damage in cases of DR. The high levels of albuminuria can increase the risk of DR. In a systematic review with metanalysis of 20 cohorts identified that age, body mass index, smoking, DR, glycaeted haemoglobin, systolic arterial pressure, high‐density lipoprotein, triglycerides and urinary albumin/creatinine ratio were variables of potential risk of diabetic nephropathy. The results also highlighted a significant superposition of factors in the causal risk between both diseases [22].

In the present study, it was observed that 27% of the participants did not know about the type of diabetes they had. It also became clear that 3% of them did not know how long they had the disease. Despite the lack of evidence of the association between DR and duration of diabetes, the prolonged exposure to hyperglycaemia has been addressed as a risk factor for progression of DR. Therefore, individual and collective actions for diabetes control in the PHC should be periodically carried out to prevent complications [23].

A higher frequency of DR was also observed in diabetic participants who were not followed up at FHU. In general, PHC is supposed to provide a longitudinal treatment of diabetic patients in which medical consultations are regularly scheduled with physician and multiprofessional team, including participation of educative groups. However, in the practice, one can perceive that these programmes are mainly aimed at issuing medical prescriptions for delivery of medicaments. Thus, it is necessary to track and monitor the DR in the context of PHC as well as of other public healthcare services for people with diabetes [24].

The implementation of methods for screening DR should be a priority in PHC because DR is well known one of the main causes of preventable blindness. In Brazil, health services have been facing challenges in conducting exams for DR regularly, mainly due to the high frequency of diabetic with people in combination with a lack of access to specialised care. A feasible alternative may be the use of teleophthalmology, which has shown to be a method facilitating such access by reducing the waiting time and, consequently, allowing early diagnosis and referral to treatment [25].

It is believed that the municipality of Jardinópolis represents the Brazilian reality either in number of habitants or in organising the public healthcare system with focus on PHC for treatment of diabetes. Therefore, it is fundamental to have a programme to control DR focused on preventing and monitoring. In view of the high demand of patients dependent on public healthcare services and since many municipalities do not have structure for providing specialise consultations, one can observe a shift to reference municipalities. In a universe of more than 12 million Brazilian individuals with diabetes, many cases progress to full blindness even before the diagnosis of DR is made or specialise treatment performed. Therefore, in the context of PHC, fundoscopy for people with diabetes could reduce by 70% the number of patients waiting for ophthalmologic consultation at specialised centres, which would allow the detection of high‐risk DR and optimise the access to proper treatment, thus decreasing the morbidity of the disease due to delayed treatment [26].

Most of the studies support the use of teleophthalmology based on images of fundus as a cost‐effective alternative to the common ophthalmologic consultation. However, there is no consensus on the calculation format to be used. Moreover, there are important variables which should be considered, such as population size, frequency of screening and cost with equipment, transportation, among others [27]. A systematic review study of the economic evidence for screening DR showed that teleophthalmology has the potential of proving efficient examinations and allowing access to remote sites. In addition, a systematic tracking is cost‐effective in terms of time with preserved vision compared to absence of screening, which allows reducing costs when DR is earlier detected and promptly treated [28].

In the present study, the low concordance between self‐reported and diagnosed DR was found to be worrisome. It was observed that the participants did not know on DR, nor how to identify signs and symptoms of the disease. Problems of refraction, glaucoma, age‐related degenerations, among others, are confounded with poor vision resulting from diabetic complications. Therefore, one can highlight the importance of education on health in the PHC through actions aimed at promoting integral care by means of nutrition strategies as well as alert signs and symptoms related to potential complications [29, 30].

## 5 Conclusions

The estimated prevalence of DR in the municipality of Jardinópolis, SP, is within the national range. The lack of knowledge on the diagnosis of DR and aspects related to the complications of diabetes were found to be worrisome. Therefore, one should emphasise the importance of programmes in the scope of public healthcare aimed to promote an integral care for diabetic people, including ocular health. Considering the Brazilian scenario, in which the small‐sized municipalities have poor intra‐structure, teleophthalmology is an alternative to be used for epidemiological surveys and models for tracking DR.

Among the study's limitations, it is mentioned that the prevalence of DR may have been overestimated, as people who used insulin were included, which generally represent a higher‐risk subset. Despite this, among the strengths of the present study, the PHC setting stands out, with a simple random probabilistic sample and data collection through interviews, blood tests, urine tests, and fundus examination with teleophthalmology, which included interaction between the academic community and public health services for this scientific production.

## Author Contributions

**Lorrana Luysse dos Anjos Assis:** conceptualisation (lead), investigation (lead), writing – original draft (lead), methodology (lead), validation (lead), visualisation (lead), writing – review and editing (lead), formal analysis (lead), data curation (lead), supervision (lead). **Fernando Korn Malerbi:** conceptualisation (supporting), investigation (supporting), funding acquisition (supporting), writing – original draft (supporting), methodology (supporting), validation (supporting), visualisation (supporting), writing – review and editing (supporting), software (supporting), data curation (supporting), supervision (supporting), resources (supporting), formal analysis (supporting). **Rosiane Chiaroti:** conceptualisation (supporting), investigation (supporting), writing – original draft (supporting), methodology (supporting), validation (supporting), visualisation (supporting), writing – review and editing (supporting), formal analysis (supporting), supervision (supporting), data curation (supporting), funding acquisition (supporting), resources (supporting). **Lívia Maria Ferrante Vizzotto Consoli:** conceptualisation (supporting), investigation (supporting), writing – original draft (supporting), writing – review and editing (supporting), visualisation (supporting), validation (supporting), methodology (supporting), formal analysis (supporting), data curation (supporting), supervision (supporting). **Vanessa Patrícia Pereira Motozo:** conceptualisation (supporting), investigation (supporting), methodology (supporting), validation (supporting), visualisation (supporting), writing – review and editing (supporting), writing – original draft (supporting), formal analysis (supporting), data curation (supporting), supervision (supporting). **Francisco Barbosa‐Junior:** conceptualisation (supporting), investigation (supporting), writing – original draft (supporting), methodology (supporting), validation (supporting), visualisation (supporting), writing – review and editing (supporting), software (supporting), formal analysis (supporting), supervision (supporting). **Rinaldo Eduardo Machado de Oliveira:** conceptualisation (lead), investigation (lead), funding acquisition (lead), writing – original draft (lead), writing – review and editing (lead), visualisation (lead), validation (lead), methodology (lead), project administration (lead), formal analysis (lead), software (lead), resources (lead), supervision (lead), data curation (lead).

## Ethics Statement

The study was approved by the Research Ethics Committee of the Dr. Joel Domingos School Health Center of the Ribeirão Preto Medical School of the University of São Paulo, following the recommendations of the National Research Ethics Commission and according to protocol number 466/2012. The Certificate of Presentation for Ethical Review was obtained with the number 64301322.0.0000.5414.

## Consent

All participants provided written informed consent at the time of entering this study.

## Conflicts of Interest

Fernando Korn Malerbi has received consulting fees from Phelcom Technologies. The other authors declare no conflicts of interest.

## Data Availability

The data that support the findings of this study are available from the corresponding author upon reasonable request.
